# Glucagon‐Like Peptide‐1 Agonists and Blood Pressure Regulation: Molecular Mechanisms and Cardiovascular Implications

**DOI:** 10.1002/prp2.70329

**Published:** 2026-10-04

**Authors:** Nora H. J. Alharbi

**Affiliations:** ^1^ Department of Pharmacology and Toxicology, College of Pharmacy University of Ha'il Ha'il Saudi Arabia

**Keywords:** blood pressure, cardiovascular outcomes, endothelial function, GLP‐1 receptor agonists, hypertension

## Abstract

Incretin effect helps in regulating glucose homeostasis and maintaining glycaemic control within the human body. This effect is achieved by the two naturally occurring incretin hormones: glucose‐dependent insulinotropic polypeptide (GIP) and glucagon‐like peptide‐1 (GLP‐1). Such hormones released by specialized entero‐endocrine cells of the gastrointestinal tract cause insulin augmentation after food consumption. However, incretin effects are impaired in type 2 diabetic, primarily attributed to the deterioration of GLP‐1 function along with decreased GLP‐1 receptor expression. Targeting this hormone has, therefore, become an important therapeutic approach to treat this diabetic condition. Native GLP‐1's brief half‐life and rapid proteolytic degradation have generated significant enthusiasm in uncovering the long‐acting GLP‐1 receptor (GLP‐1R) agonists to manage type 2 diabetes. These synthetic peptides mimic the natural GLP‐1 to activate GLP‐1R and effectively exert various physiological benefits in terms of weight management, blood pressure (BP), lipid profile, obesity and major adverse cardiovascular event. Although the BP‐lowering effect of GLP‐1R agonists is well documented in clinical trials, the underlying molecular mechanisms remain incompletely defined, since most evidence comes from animal and in vitro models rather than human studies. This gap limits translating the clinical benefit into targeted, mechanism‐based practice. This review paper provides insights into the structure, function and expression of GLP‐1 and its receptor. It also summarizes existing literature regarding different types of GLP‐1R agonists, their effects on BP and how they exert their actions. Evidence indicates GLP‐1R agonists produce a modest, consistent reduction in systolic blood pressure, heterogeneous across agents and confined to patients with elevated baseline BP, reflecting vascular, renal and anti‐inflammatory mechanisms rather than a single pathway. Although animal models have contributed significantly to this area of study, the scarcity of human studies is noted, indicating a need for more investigation in this domain. Expanding research in humans will help in comprehending the impact of GLP‐1R agonists on BP regulation and their underlying mechanisms.

## 1 Introduction

Plasma glucose serves as the primary metabolic fuel for the central nervous system (CNS) [1]. Brief episodes of hypoglycaemia can lead to significant CNS dysfunction while prolonged and severe hypoglycaemia may result in cellular damage or death. To prevent hypoglycaemia and to maintain proper functioning of various metabolic processes, the human body has therefore evolved a regulatory mechanism that tightly controls blood glucose levels. Because of this system, a healthy individual possesses a remarkable capacity to manage increasing glucose intake and maintain nearly unchanged postprandial (after meal) glucose excursions, regardless of the oral load of glucose consumed [2, 3]. The incretin effect is one such regulatory mechanism responsible for glucose homeostasis within the human body. Due to this effect, the blood sugar level is regulated by the augmentation of insulin secretion (by a factor of 2–3) from beta cells of the pancreas after oral intake of glucose as opposed to intravenous infusion [4]. This incretin effect is achieved by two types of incretin hormones released from specialized entero‐endocrine cells, namely glucose‐dependent insulinotropic polypeptide (GIP) and glucagon‐like peptide‐1 (GLP‐1) [5]. Among these, GLP‐1 receives much attention because it not only possesses glucose‐lowering effects but also demonstrates its capability to delay the gastric emptying process and suppress glucagon secretion [6]. In addition, type 2 diabetic patients show reduced levels of this incretin effect as a result of impaired insulin secretion, leading to hyperglycaemia. This deficit is attributed to the deterioration of GLP‐1 function along with decreased GLP‐1 specific receptor expression [7, 8]. Given its various physiological effects, GLP‐1 emerges as an attractive candidate for therapeutic interventions for managing type 2 diabetes. Its multifunctional nature offers potential benefits for addressing various aspects of the condition other than its glycaemic control. Although several reviews have discussed the metabolic and cardiovascular benefits of glucagon‐like peptide‐1 receptor agonists, a focused synthesis of their role in blood pressure regulation remains limited. Unlike previous reports, the present mini‐review specifically integrates molecular, vascular, renal, and clinical evidence linking GLP‐1 receptor activation to blood pressure modulation. Particular emphasis is placed on discrepancies between animal and human findings, drug‐specific heterogeneity, and the clinical relevance of small but consistent reductions in systolic blood pressure. This targeted approach aims to clarify mechanistic pathways and identify critical knowledge gaps relevant to cardiovascular risk reduction.

Accordingly, the specific objectives of this review are as follows: first, to summarize the molecular mechanisms and intracellular signaling pathways through which GLP‐1 receptor activation affects vascular, renal, and cardiac function. Second, to critically evaluate the clinical trial evidence describing the magnitude and consistency of blood pressure change associated with individual GLP‐1 receptor agonists. Finally, to underline the discrepancies between preclinical and human data that currently limit our mechanistic understanding in order to inform future translational research.

## 2 Approaches to Literature Review

A literature review was conducted using PubMed, Scopus, and Web of Science databases. Articles were classified using various combinations of keywords: GLP‐1 receptor agonists, glucagon‐like peptide‐1, blood pressure, hypertension, cardiovascular outcome, and vascular function. The review was limited to research articles, clinical trials, meta‐analysis, and reviews published in the English language. The emphasized research illustrated the capability of GLP‐1 receptor agonists to regulate blood pressure in both human and animal models. Articles that did not particularly mention cardiovascular mechanisms or blood pressure were excluded.

## 3 Glucagon‐Like Peptide 1 (GLP‐1)

### 3.1 Structure

Among GIP and GLP‐1, GLP‐1 holds great promise as an incretin hormone. This hormone exists in various forms like GLP‐1 (1–37), GLP‐1 (1‐36amide), GLP‐1 (7–36amide), GLP‐1 (7–37), and their efficacy in boosting insulin secretion when glucose is present appears to vary among these forms [9]. Of these forms, GLP‐1 (7–36amide) and GLP‐1 (7–37) are recognized as truncated versions of this hormone and are called amidated GLP‐1 and glycine‐extended GLP‐1, respectively [10]. The distribution of various types of GLP‐1 differs among different species [11]. In humans, the majority of the circulating GLP‐1 are truncated ones, of which approximately 80% of them are GLP‐1 (7–36amide), whereas only a few percentage (about 20%) corresponds to GLP‐1 (7–37), as reflected from immunoreactivity data [12].

### 3.2 Synthesis

GLP‐1 is synthesized in the intestine from the proglucagon composed of 180‐amino acids as a result of post‐translational modifications [9]. After its formation, prohormone convertase 1 (PC1) cleaves proglucagon to produce 37‐amino acid immature GLP‐1 along with GLP‐2, glicentin, oxyntomodulin and intervening peptide‐2. This immature GLP‐1 is then rapidly modified into its active forms, that is, GLP‐1 (7–36) amide and glycine‐extended GLP‐1 (7–37) as reported in rodents and humans [13].

### 3.3 Secretion

Enteroendocrine L‐cells, that is present in ileum of distal part and colon, releases GPL‐1 following the consumption of carbohydrate and non‐carbohydrate foods [14]. Upon metabolism, glucose as well as fructose induce the secretion of GLP‐1 by closing ATP‐dependent K‐channels. This closure induces membrane depolarization that releases voltage‐dependent calcium channels (L‐type) in the intestine [13]. Opening of these channels results in Ca^2+^ influx inside enteroendocrine GLUTag cells and causes GLP‐1 to secrete into the blood through vesicular exocytosis [15]. Stimulation of GLP‐1 secretion is also caused by free fatty acids (FFAs) through various mechanisms. Both short and long‐chain FFAs bind to their respective fatty acid receptors such as GPR43, GPR120 [16] and causes calcium ions to enter through Ca^2+^ channels (most likely L‐type) present on the cell surfaces [17]. This influx subsequently increases the concentration of intracellular calcium ions, thereby inducing GLP‐1 release. A receptor‐independent mechanism regarding the secretion of GLP‐1 is also evidenced as a result of uncoupling oxidative phosphorylation and glycolysis activation [18]. Protein intake can also lead to the release of GLP‐1 through the activation of Ca^2+^/calmodulin‐dependent kinase II [19] and transmission of signals to L‐cells via peptide transporter 1 (PEPT1) and Ca^2+^ sensing receptor (CaSR) [20].

### 3.4 Metabolism

The two active forms of GLP‐1, namely, GLP‐1 (7–36) amide and the glycine‐extended GLP‐1 (7–37) have a brief survival duration as they are converted into their inactive forms by a proteolytic enzyme at a faster rate [21]. As soon as they are released from the L‐cells of the intestine, around 33% of GLP‐1 (7–36) amides are truncated immediately at the site of secretion into the inactive form, that is, GLP‐1 (9–36) amide [22] whereas the rest 43% molecules are degraded subsequently in the hepatocyte [23]. This rapid degradation leads to only 38% of GLP‐1 to remain active. They are secreted from the intestine to enter the circulatory system where they are further subjected to proteolytic degradation. Consequently, only a few active molecules are destined to reach their targets [14, 23] where they can bind with its receptor (GLP‐1R) to induce its biological effects [24]. The binding is followed by the recognition of the ligand mediated by the specific residues present in extracellular domain at the N‐terminal region of GLP‐1R interacting with the α‐helical region of the ligand [25, 26]. Following this, the N‐terminal residues of ligand binds with the extracellular loops and transmembrane helices of the receptor to induce the activation of GLP‐1R [27, 28, 29].

## 4 Glucagon‐Like Peptide 1 Receptor (GLP‐1R)

### 4.1 Structure

GLP‐1 receptors (GLP‐1R) belong to the class B type of G protein‐coupled receptors (GPCRs) [30]. The GLP‐1R protein of humans comprises 463 amino acids [9]. Immediately after its synthesis in the endoplasmic reticulum (ER), GLP‐1R is translocated across the ER with the help of a signal peptide coded by its short leader sequence [31]. Following translocation, this signal peptide is removed by a peptidase, giving rise to a complete structure of this protein. It has the α‐helix at the N‐terminal region of the extracellular domain and four β‐strands arranged in two sheets antiparallelly. Three disulfide bonds connect these two sheets involving six cysteine (Cys) residues [29]. The first disulfide bond between the first and third Cys residues links the α‐helix to the first β‐sheet, whereas the second bond between the second and fifth Cys residues connects two β‐sheets. At last, the third one is formed between the fourth and sixth residues which brings the central β‐sheets in close proximity to the GLP‐1R's C‐terminal domain [9].

### 4.2 Expression

Attempts have been made to elucidate the expression pattern of this receptor across various species such as in rats, mice, monkeys, and humans. Findings revealed the expression of GLP‐1R in various regions including the lungs, gastrointestinal tract, pancreas, kidneys, and CNS in all these experimental models [9, 13]. However, the precise locations of this expression within these organs exhibited variations among the species. In the heart, its presence is reported in mice, monkeys, and humans. Interestingly, GLP‐1R does not express within the liver of rats, monkeys, and humans. In mice, although GLP‐1R expression is not present in hepatocytes, it is found in the portal vein walls situated close to the liver hilum [32].

### 4.3 Metabolism

GLP‐1R can exist in both active and inactive forms which is determined by its interaction with the type of allosteric modulators. Binding of a negative allosteric modulator within the cleft formed by helices VI and VII of GLP‐1R blocks the association of helix VI with the G protein, leading to the deactivation of the receptor [33]. Conversely, the positive allosteric modulator binds between helices V and VI and activates the receptor by facilitating the G‐protein binding with the GLP‐1 receptor. Once activated, the receptor triggers several signaling pathways including Gs signaling for cAMP formation, Gq/11 pathway for elevating intracellular Ca^2+^ concentration and ERK1/2 signaling for recruiting β‐arrestin [34, 35, 36]. Although various GLP‐1 forms can bind with the same receptor, each of them initiates signaling pathways different from the other and elicits diverse cellular responses [36, 37]. In the pancreas, activated GLP‐1 receptor stimulates the production of insulin through adenylyl cyclase pathway which is subsequently released into the bloodstream, aiding in regulating the plasma glucose level [38].

### 4.4 Signaling Pathways Linking GLP‐1 Receptor Activation to Cardiovascular Regulation

Beyond the Gs‐cAMP‐PKA and Gq/11‐Ca^2+^ signaling that GLP‐1R engages in the pancreas, this receptor recruits several additional intracellular cascades that are directly relevant to cardiovascular and renal function. In vascular endothelial cells, GLP‐1R activation recruits the phosphatidylinositol 3‐kinase (PI3K)/Akt pathway, leading to phosphorylation and activation of endothelial nitric oxide synthase (eNOS) and a consequent rise in nitric oxide (NO) bioavailability, an effect demonstrated directly in human umbilical vein endothelial cells exposed to GLP‐1 [39]. This PI3K‐Akt‐eNOS axis operates alongside protein kinase A (PKA)‐dependent NO production, providing two converging routes by which GLP‐1R stimulation can relax vascular smooth muscle and reduce peripheral resistance. In the heart, GLP‐1R activation on atrial cardiomyocytes couples to the exchange protein activated by cAMP 2 (Epac2), triggering secretion of atrial natriuretic peptide (ANP); the resulting rise in cyclic guanosine monophosphate (cGMP) promotes both vasodilation and renal sodium excretion [40].

At the receptor level, biased agonism means that different GLP‐1R ligands can preferentially engage Gs/cAMP, Gq/11/Ca^2+^, or beta‐arrestin/ERK1/2‐linked signaling to differing degrees, which may partly explain why structurally distinct GLP‐1 receptor agonists do not lower blood pressure to the same extent responses [36]. Downstream of these proximal signaling events, activation of AMP‐activated protein kinase (AMPK) and suppression of nuclear factor‐kappa B (NF‐kB) signaling converge to reduce vascular oxidative stress, adhesion molecule expression and inflammatory cytokine release [41, 42], linking the acute vasoactive signaling of GLP‐1R to the slower anti‐atherosclerotic remodeling of the vessel wall discussed later in this review. Taken together, these pathways indicate that the blood pressure‐lowering potential of incretin‐based therapies is not attributable to a single mechanism, but rather to the convergence of vascular, renal, cardiac and anti‐inflammatory signaling downstream of a single receptor (Figure 1).

**FIGURE 1:**
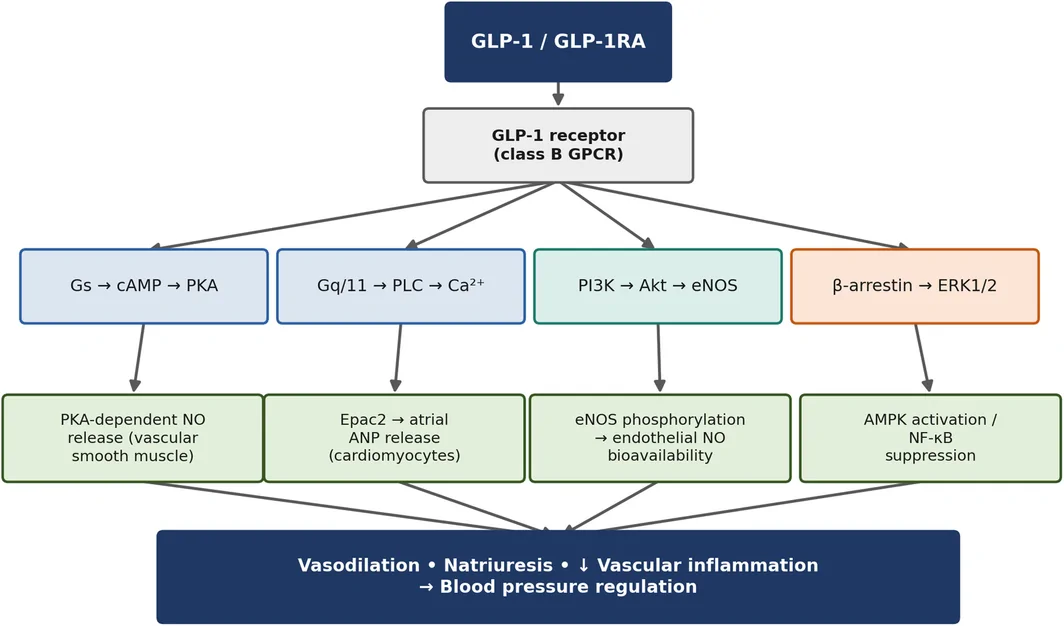
Overview of GLP‐1 receptor intracellular signaling pathways relevant to cardiovascular and blood pressure regulation.

## 5 Glucagon‐Like Peptide 1 Agonists (GLP‐1RAs)

Native GLP‐1 acts as a multifaceted hormone that exhibits a plethora of physiological activities in the body [9]. It primarily has the potential to reduce hypoglycaemia for which it has become an attractive candidate for pharmacists to manage patients having type 2 diabetes mellitus. Unfortunately, this hormone has a very short life‐span (half‐life of 1.5 min) as it undergoes rapid degradation by the dipeptidyl peptidase‐4 (DPP‐4) enzyme [43]. In order to achieve the therapeutic benefits of the native GLP‐1, it is necessary to administer GLP‐1 in patients continuously throughout the day [44] which is impossible and therefore restricts its therapeutic use for type 2 diabetes. To overcome these issues, several drugs are being innovated and designed aiming to cure type 2 diabetes therapeutically. Among them, derivatives of native GLP‐1 with an increased half‐life, in contrast to natural GLP‐1, are given much importance for their ability to produce prolonged therapeutic effects. GLP‐1 receptor agonists (GLP‐1RA) are a type of medication that falls into this category. They are designed in such a way that they mimic the natural hormone but are not sensitive to enzymatic ubiquitination [45]. Hence, GLP‐1RA has been considered an important target in treating type 2 diabetes in recent years.

Although designing GLP‐1RAs, it is desirable to minimize unnecessary alterations in amino acids in these analogs and maintain structural similarity between the analogs and native GLP‐1 to avoid immunogenicity responses [46]. Several GLP‐1RAs are nowadays available in the market which are categorized into short‐ and long‐acting groups [47, 48]. Short‐acting agents comprise exenatide twice daily as well as lixisenatide, whereas exenatide weekly once, albiglutide, liraglutide, semaglutide, and dulaglutide belong to long‐lasting groups.

Exenatide is a mimetic of exendin‐4 that was originally derived from the saliva of *Heloderma suspectum* , also known as the Gila monster [49]. This isolated peptide analog shows more than 50% similarity with the sequence of native GLP‐1 and possesses a half‐life of around 2.4 h [50]. This compound was first approved in 2005 for its twice daily administration [51]. Liraglutide closely resembles human GLP‐1 and carries a fatty‐acid side chain, attached via a glutamic acid spacer, that promotes reversible, non‐covalent binding to human serum albumin [52, 53]. It has an increased half‐life, that is, 8–10 h after its administration intravenously and 13–15 h after subcutaneous administration [52, 54, 55]. Hence, this analog is approved in 2009 for its once‐daily administration [56] whereas GLP‐1RAs such as, dulaglutide, albiglutide, and semaglutide are aimed for once‐weekly administration. Albiglutide comprises two Gly8 GLP‐1 molecules fused with serum albumin of human in tandem order. This RA has a half‐life of around 6–8 days and takes 2–4 days to reach the maximum plasma concentration (*T*_max_) [57]. Recently, dulaglutide was accepted as a second analog for its once‐weekly administration. Regarding semaglutide, substitutions are made at two amino acids in relation to human GLP‐1 and is derivatized at lysine 26 [46]. This analog has approximately half‐life of 1 week, allowing for its once‐weekly subcutaneous administration. Apart from these, taspoglutide was also formulated as a long‐acting GLP‐1 RA but never reached commercialization due to challenges with its tolerability that significantly affected the cardiovascular outcome trial (CVOT) linked to its development [9]. Efpeglenatide is another member of the long‐acting group which contains an altered exendin linked to the human immunoglobulin 4 fragment using an advanced technology [58, 59]. This conjugation increases the duration of efpeglenatide's action [60] and the short flexible polyethylene glycol based linker helps to maintain its intrinsic activity. Currently, this RA is undergoing development with the aim to regulate glycaemia in patients suffering from type 2 diabetes through their sub‐cutaneous administration once‐weekly. Tirzepatide is another incretin mimetic which functions as a dual receptor agonist for both GIP and GLP‐1 [61]. This synthetic peptide is composed of 39 amino acids and is formulated on the basis of the sequence of native GIP [62]. Both clinical and pre‐clinical trials have demonstrated promising results regarding glucose reduction, weight management and effects on hemoglobin A1c (HbA1c) along with some adverse effects. Its impact on cardiovascular outcomes, long‐term effectiveness and safety‐profile have not been established and are currently under investigation.

Short‐acting agonists for GLP‐1R have a remarkable ability to lower glucose levels following meal consumption (postprandial) and delay gastric emptying. In contrast, long‐acting agents show glucose‐lowering effects on not only postprandial glucose levels but also on fasting glucose levels. This regulation of glucose levels is achieved by stimulating (glucose‐dependent) insulin secretion while inhibiting glucagon release [47]. Moreover, these long‐acting agonists cause smaller variations in fluctuations in drug concentrations of plasma and improve tolerability of gastrointestinal profiles. Due to their simplicity and convenience in terms of administration schedules, long‐acting RAs increase treatment adherence and persistence in patients, thereby improving their clinical outcomes. Recent clinical and pharmacological advances have continued to expand this treatment landscape including newer agents and combination approaches [63].

## 6 Effects of GLP‐1RAs on Blood Pressure

Among all known GLP‐1R agonists, utilization of albiglutide, dulaglutide, semaglutide, and liraglutide as a new treatment option for diabetic patients in pharmaceutical industries gains more popularity. They are safe in effectively controlling blood glucose levels without causing hypoglycaemia upon administration. Additionally, these RAs are reported to lower occurrences of major adverse cardiovascular events (MACE) associated with diabetes such as stroke, acute coronary syndrome, myocardial infarction, heart failure, and cardiovascular death substantially [61, 64]. Evidences from cardiovascular outcome trials have shown that GLP‐1RAs play crucial roles in modifying several risk factors of cardiovascular diseases including blood pressure, obesity, lipid profile and body weight that contribute significantly in the observed cardioprotective effects among diabetic population. The present review focuses only on the impacts of GLP‐1RAs specifically on blood pressure, along with their mechanisms behind these beneficial effects in both diabetic as well as non‐diabetic subjects.

### 6.1 Clinical Outcomes of Different GLP‐1RAs on Blood Pressure

Hypertension is found to be one critical risk factor that causes cardiovascular diseases. Because diabetes mellitus (DM) coexists with arterial hypertension at a higher rate, effectively managing blood pressure becomes crucial in handling patients suffering from DM to prevent their health complications or mortality [65, 66]. The clinical trials on experimental models of hypertension demonstrated that GLP‐1 RAs can potentially lower the blood pressure [67, 68], particularly systolic blood pressure (SBP) with 2–3 mmHg of average reduction [69, 70]. However, diastolic blood pressure (DBP) either remains unchanged [71, 72] or shows no significant impact [73] upon administering agonists for GLP‐1 receptors.

Administration of exenatide at 10 μg twice daily appeared to ameliorate SBP (least squares mean difference was 2.8 mmHg) in comparison to insulin or placebo, as derived from a pooled analysis encompassing data involving 2171 subjects from six trials [74]. The reduction of SBP was most significant between exenatide and placebo (difference of 8.2 mmHg) among subjects whose baseline SBP was ≥ 150 mmHg. However, those people having normal BP level at the baseline showed no between‐group differences in the alteration of SBP or DBP between the two groups (exenatide and placebo) [74]. Upon administering a subcutaneous injection of 2 mg exenatide once‐weekly to 14 752 patients having T2DM, the least squares mean for SBP values was reduced (1.6 mmHg) at 6 months when compared with patients injected with the placebo [75]. Another randomized comparator‐controlled trial involving 1379 individuals demonstrated notable reductions in SBP and DBP by 2.8‐ and 0.8‐mmHg, respectively in patients after treated with exenatide once‐weekly for 24–30 weeks [76].

On the other hand, dulaglutide at 1.5 mg resulted in the reduction of SBP (2–3 mmHg) than the placebo, which was evidenced by 4 weeks and remained consistent throughout 26 weeks [77]. Along with this, dulaglutide was able to reduce the mean 24‐h pulse pressure in these patients.

The effect of lixisenatide on the reduction of BP was also evaluated in a randomized placebo‐controlled (ELIXA) trial. This trial randomly assigned 6068 patients diagnosed with T2DM who had recently experienced an acute coronary syndrome to receive either lixisenatide or placebo. Throughout the 40‐month follow‐up period, a slight decrease in SBP of 0.8 mmHg was observed and sustained within the lixisenatide group, unlike in the placebo group [78].

In another randomization trial (SUSTAIN‐6), nearly 3297 patients diagnosed with T2DM were administered semaglutide once‐weekly at two different (0.5 and 1.0 mg) doses [72]. Compared to the placebo, semaglutide at 1.0 mg only was able to significantly lower the average SBP by 2.6 mmHg while no significant reduction was observed with semaglutide 0.5 mg. When semaglutide was taken orally once daily at a dosage of 14 mg, patients having type 2 diabetes and prone to cardiovascular issues had lower SBP by 2.6 mmHg at Week 83 [73]. A similar lowering effect of liraglutide on SBP was noticed in a comparable patient population in the double‐blind (LEADER) trial [71]. In this case, SBP was decreased by 1.2 mmHg from baseline to 36 months.

The collected evidence from clinical trials and meta‐analyses indicates that various types of GLP‐1 RAs can notably decrease blood pressure in diabetic patients with or without cardiovascular risks but at different levels. However, these RAs do not demonstrate such lowering effect in normotensive subjects or individuals showing normal blood pressure [79] (Table 1).

**TABLE 1:** Effects of GLP‐1 receptor agonists on blood pressure in human studies.

GLP‐1RA	Dose/regimen	Study type	Population	SBP change (mmHg)	DBP change (mmHg)	Key references
Exenatide	10 μg, twice daily	RCT, pooled analysis	T2DM	−2.8	No significant change	Okerson et al. [74]
Exenatide	2 mg, once weekly	CVOT (EXSCEL)	T2DM with CV risk	−1.6	Not significant	Holman et al. [75]
Dulaglutide	1.5 mg, weekly	RCT	T2DM	−2 to −3	Minimal	Ferdinand et al. [77]
Lixisenatide	20 μg, daily	CVOT (ELIXA)	T2DM + ACS	−0.8	No change	Pfeffer et al. [78]
Semaglutide	1.0 mg, weekly	CVOT (SUSTAIN‐6)	T2DM	−2.6	Not significant	Marso et al. [71, 72]
Semaglutide	14 mg, oral	CVOT (PIONEER‐6)	T2DM	−2.6	Not significant	Husain et al. [73]
Liraglutide	Up to 1.8 mg, daily	CVOT (LEADER)	T2DM with CV risk	−1.2	No change	Marso et al. [71, 72]

### 6.2 Mode of Action

Before discussing these mechanisms, it is important to differentiate between the acute and chronic cardiovascular effects of GLP‐1 receptor agonists, as the literature often mixes between them. Acute stimulation of GLP‐1 receptors causes a small but reproducible increase in heart rate, mediated by direct stimulation of GLP‐1 receptors on sinoatrial node pacemaker cells [80] as well as by central stimulation of brainstem autonomic neurons which increase sympathetic outflow [81]. This acute chronotropic response is accompanied by a small, transient increase in mean arterial pressure, particularly in the hours following dosage escalations. This is contrary to the chronic, steady‐state effects of GLP‐1 receptor agonists described throughout this section, in which sustained therapy is associated with a net reduction in systolic blood pressure. The chronic reduction is the result of the cumulative effect of weight loss, improved glycaemic control, and the vascular, renal and anti‐inflammatory mechanisms discussed below, which together outweigh the initial, sympathetically mediated pressor response when treatment is continued [82]. This acute vs. chronic difference is clinically relevant as it explains why some patients have a transient increase in heart rate soon after starting or increasing therapy, and this is not incompatible with the blood pressure benefit that develops over weeks to months of continued use (Figure 2).

**FIGURE 2:**
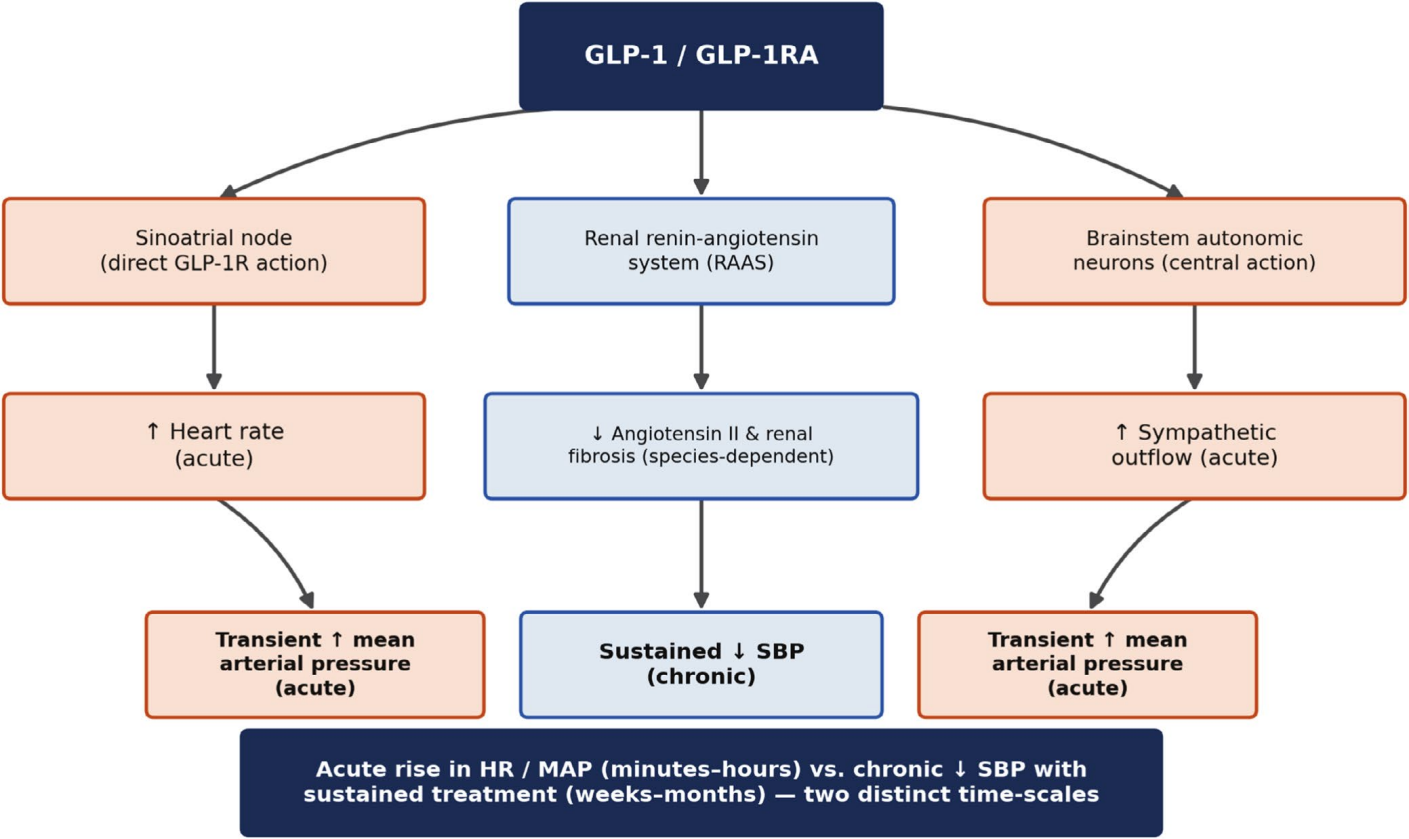
Acute central/sinoatrial actions of GLP‐1 receptor agonists versus their chronic modulation of the renin‐angiotensin system.

According to Ferdinand et al. [77], patients who were treated with dulaglutide and showed reduction in SBP did not exhibit significant alterations in plasma renin activity, serum aldosterone, plasma meta‐nephrines, normetanephrines, and N‐terminal pro‐brain natriuretic peptides. As a result, the authors proposed that dulaglutide probably lowers blood pressure in these patients through some mechanisms that are not related to neurohormonal and renin–angiotensin–aldosterone systems. On the contrary, Le et al. [83] established that exendin‐4 significantly reduced levels of angiotensin (Ang) II within the kidney of mice. It also inhibited the signaling pathway involving Ang II that mediates transforming growth factor (β1) and Smad3. These data showed that exendin‐4 has the ability to modulate the renin‐angiotensin system in mice. These discrepancies in experimental findings have resulted in inconclusive reports of the impact of GLP‐1 RAS on the renin–angiotensin system, which prompts the need for further investigation to elucidate the relationship between the two (Table 2).

**TABLE 2:** Proposed mechanisms of blood pressure regulation by GLP‐1 receptor agonists.

Mechanism	Target tissue	Evidence type	Key findings	References
Vasodilation	Vascular endothelium	Animal & human	Improved NO bioavailability and reduced vascular tone	Liu et al. [84]
Endothelial function	Endothelium	Animal	Reduced oxidative stress and improved endothelial function	Gaspari et al. [85]
Natriuresis	Kidney	Human	Increased sodium excretion contributing to BP reduction	Lovshin et al. [86]
ANP release	Cardiac atria	Animal	GLP‐1R activation stimulates ANP	Kim et al. [40]
RAAS modulation	Kidney	Animal	Reduced Ang II signaling and fibrosis	Le et al. [83]
Anti‐inflammatory effects	Vascular wall	Animal & in vitro	NF‐κB inhibition and reduced adhesion molecule expression	Helmstädter et al. [41]

It is reported that GLP‐1 RAs can induce natriuresis among healthy, obese and T2DM individuals, potentially contributing to the reduction of blood pressure [86]. According to Kim et al. [40], liraglutide stimulates its receptor to secrete atrial natriuretic peptide (ANP) from atrial cardiomyocytes in male mice treated with Ang II which triggers various effects including vasodilation mediated by cyclic guanosine monophosphate (cGMP), improved permeability of endothelium and excretion of sodium from the renal system [40]. Although antihypertensive effects of GLP‐1 RAs against hypertension through ANP release were found in animals, such mechanism has not been fully elucidated in humans [86, 87]. These renal and cardiac natriuretic pathways are summarized in Figure 3.

**FIGURE 3:**
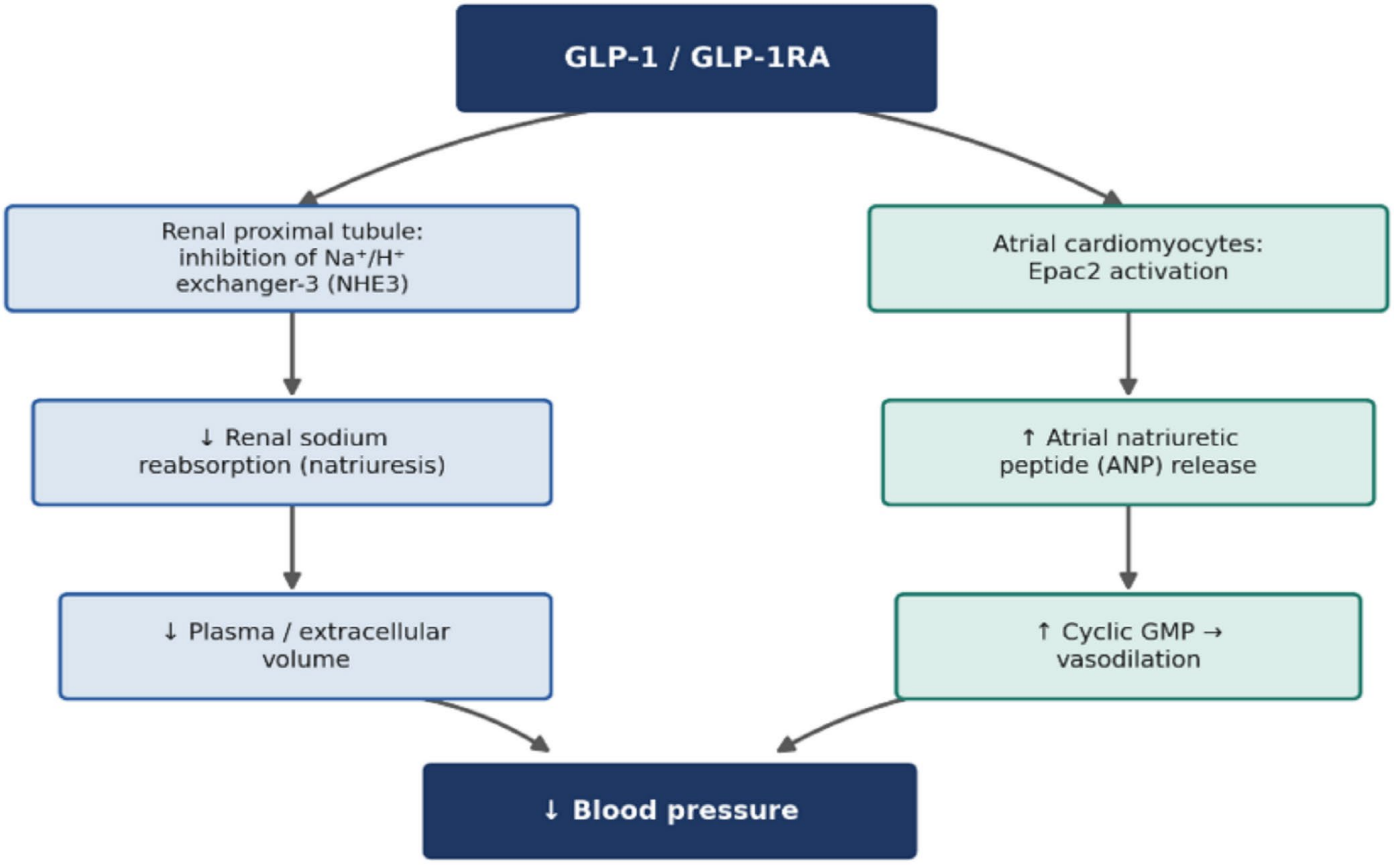
Renal natriuretic and cardiac ANP/Epac2‐mediated pathways contributing to blood pressure lowering by GLP‐1 receptor agonists.

Existing research has indicated that GLP‐1 RAs reduce SBP by their ability to induce vasodilation within the arterial vasculature and decrease vascular contraction [84, 88]. The function of the endothelium is also enhanced, which can also aid in normalizing blood pressure [89]. Murine models subjected to liraglutide treatment improved endothelial functions [85]. GLP‐1 analogs inhibit inflammation and oxidative stress, particularly in individuals diagnosed with diabetes mellitus (Type 1), thereby mitigating the impairment of endothelial functions [90]. Additionally, Liu et al. [84] showed that endothelial cell functions are improved by triggering nitric oxide (NO) release and reducing oxidative stress, thereby regulating vascular contractions. Helmstädter et al. [41] observed that liraglutide could downregulate the NF‐κB signaling pathway and effectively suppress the inflammatory cascade induced by angiotensin II in vascular walls. Consequently, several adhesion molecules such as ICAM‐1, P‐selectin, VCAM‐1 could not express on the surface of endothelial cells, which diminishes vascular oxidative stress and restores NO levels. Because NO has a pivotal role in preserving vascular homeostasis [91], mice treated with liraglutide therefore had a lower risk of endothelial dysfunction induced by hypertension and angiotensin II [41]. This endothelial nitric oxide‐mediated vasodilatory pathway is illustrated in Figure 4.

**FIGURE 4:**
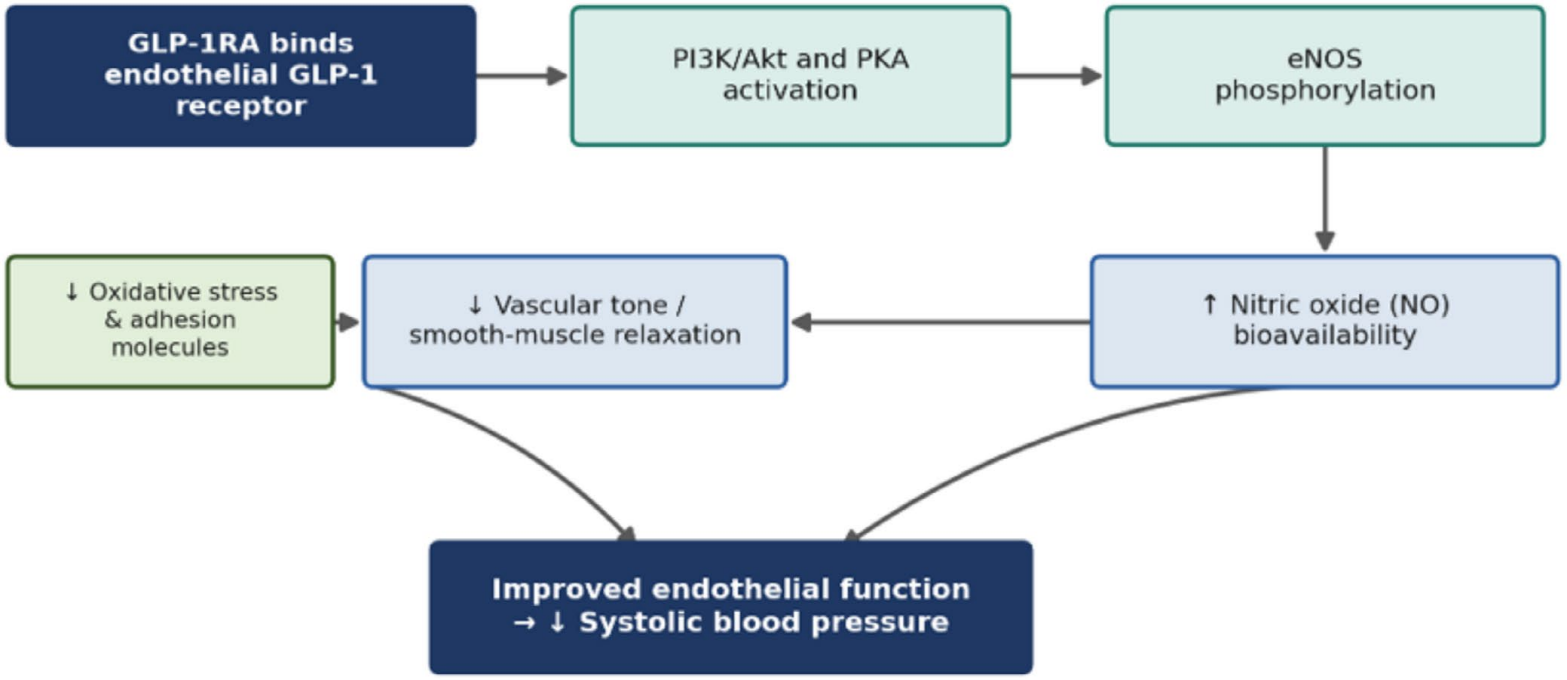
Endothelial nitric oxide‐mediated vasodilatory pathway underlying the antihypertensive effect of GLP‐1 receptor agonists.

Upon administration, liraglutide was reported to diminish vascular inflammation and curb neointimal hyperplasia in another study, which is achieved through the enhancement of nitric oxide bioavailability [92]. Development of atherosclerosis within vascular walls has a notable influence on blood pressure regulation [61]. This process encompasses the interaction between matrix metalloproteinases (MMPs) and tissue inhibitors of metalloproteinases (TIMPs), which significantly influences the development of atherosclerosis [93]. The expression of these two proteins in both endothelial cells and smooth muscle cells within the coronary artery in humans were found to be modulated by exenatide via inhibition of the NF‐kB and Akt signaling pathways [94, 95], thereby indirectly regulating blood pressure. In addition, it was observed that GLP‐1 RAs are able to diminish precursors of atherosclerotic disease, that is, vascular adhesion molecules in vitro [85]. Researches have shown that liraglutide has the ability to decrease the elevation of vascular adhesion molecules, specifically vascular cell adhesion molecule‐1 (VCAM‐1), intercellular adhesion molecule‐1 (ICAM‐1), and E‐selectin in endothelial cells after treatment, potentially by activating signaling pathways involving AMP‐activated protein kinase (AMPK) and calcium/calmodulin‐dependent protein kinase I. Moreover, this GLP‐1RA exhibits the capacity to reduce Endothelin‐1 (ET‐1)'s activity which tends to get upregulated in the endothelium during atherosclerosis and constricts the blood vessels [96]. This effect might in turn lower the activity of NF‐kB. On the other hand, the phenotypic change of macrophages from M1 to M2 induced by exenatide not only reduces adverse alterations in the vascular wall but also stabilizes atherosclerotic lesions [97]. These actions collectively indicate the potential of GLP‐1RAs in mitigating factors involved in vascular inflammation and vasoconstriction to manage atherosclerosis‐related changes. The administration of semaglutide or liraglutide to mice reduced the development of plaques during atherosclerosis [98], possibly due to the increase in the levels of AMPK intracellularly [42] and decrease in proinflammatory cytokines, including NF‐κB, tumor necrosis factor or TNF‐α and monocyte chemoattractant protein‐1 in cells treated with GLP‐1RAs [99]. These alterations in cellular responses provide insights into the probable mechanisms through which GLP‐1RAs could mitigate atherosclerosis development. Anti‐inflammatory and anti‐atherosclerotic pathway is summarized in Figure 5.

**FIGURE 5:**
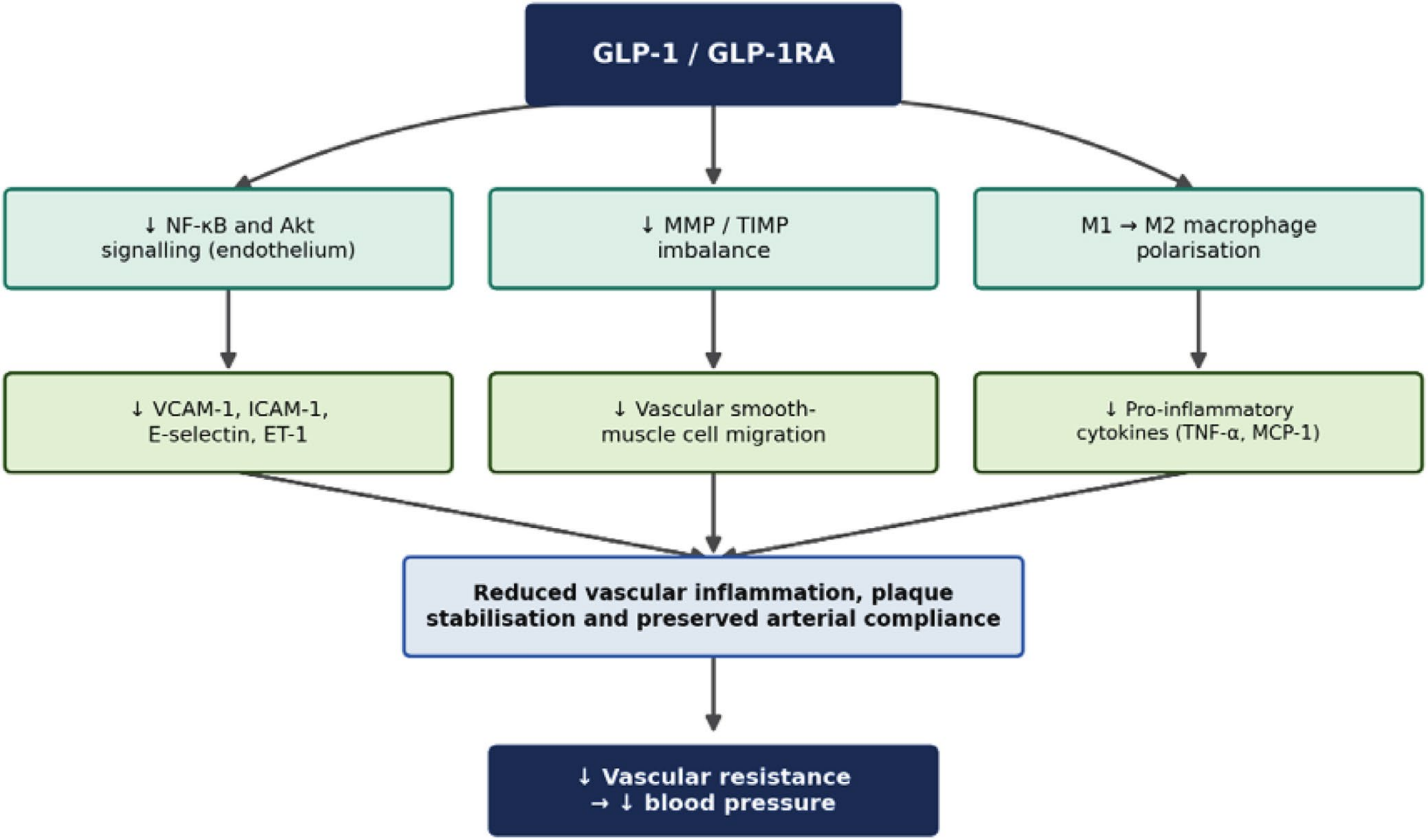
Anti‐inflammatory and anti‐atherosclerotic actions of GLP‐1 receptor agonists contributing to long‐term vascular resistance and blood pressure lowering.

### 6.3 Knowledge Gaps and Future Directions

Despite growing evidence that glucagon‐like peptide‐1 agonists induce uncertain reductions in systolic blood pressure, the clinical relevance of these effects remains incompletely understood. Most available data originate from cardiovascular outcome trials where blood pressure was not a primary endpoint, making it difficult to determine whether the observed reductions translate into meaningful antihypertensive benefits independent of glycaemic control or weight loss.

In addition, mechanistic insights into blood pressure regulation by GLP‐1 receptor agonists are mainly derived from animal and in vitro studies. Human evidence supporting proposed pathways such as natriuresis, endothelial nitric oxide signaling, atrial natriuretic peptide release, and modulation of the renin‐angiotensin system remains limited and sometimes inconsistent, highlighting the need for translational validation. The major knowledge gaps, limitations, and future research directions regarding the blood pressure‐lowering effects of GLP‐1 receptor agonists are summarized in Table 3.

**TABLE 3:** Current limitations and future research directions.

Issue	Current evidence	Limitation	Suggested future research
Modest BP reduction	SBP ↓ 1–3 mmHg	Clinical significance unclear	Large BP‐focused trials
Human mechanism data	Limited	Mostly animal evidence	Translational human studies
DBP effects	Inconsistent	Underreported	Standardized BP assessment
Drug‐specific effects	Heterogeneous	Head‐to‐head data lacking	Comparative clinical trials
Normotensive patients	No BP benefit	Mechanism unknown	Stratified patient studies

## 7 Conclusion

This review synthesizes the current evidence regarding the effect of glucagon‐like peptide‐1 receptor agonists on blood pressure, a relationship that is clinically important but mechanistically incomplete. Across cardiovascular outcome trials and randomized studies, GLP‐1 receptor agonists as a class produce a small, generally consistent reduction in systolic blood pressure, ranging from under 1 mmHg with lixisenatide to approximately 2–3 mmHg with exenatide and dulaglutide, whereas diastolic blood pressure is largely unaffected. This benefit appears to be confined to individuals with elevated baseline blood pressure and is not observed in normotensive subjects, suggesting a regulatory rather than a purely pharmacological action.

Mechanistically, this effect cannot be attributed to any single pathway. Instead, it appears to arise from the convergence of several processes downstream of GLP‐1 receptor activation: PI3K‐Akt‐eNOS‐ and PKA‐mediated nitric oxide release improving endothelial vasodilation; Epac2‐dependent atrial natriuretic peptide secretion and direct renal natriuresis reducing plasma volume; variable modulation of the renin‐angiotensin system; and AMPK‐mediated, NF‐kB‐dependent suppression of vascular inflammation and adhesion molecule expression that slows atherosclerotic progression. These chronic, blood pressure‐lowering actions must be distinguished from the acute chronotropic and mildly pressor effect that follows GLP‐1 receptor stimulation of the sinoatrial node and brainstem autonomic centers; the two operate on different time‐scales and are not contradictory, although conflating them has likely contributed to inconsistency in the literature.

A recurring limitation is that most of this mechanistic evidence, with the exception of the natriuretic and endothelial function data, is derived from animal and in vitro systems rather than confirmed in humans, and blood pressure has rarely been a pre‐specified primary outcome in the major cardiovascular outcome trials. Future research should prioritize blood pressure as a primary endpoint, pursue mechanistic studies in human subjects, and directly compare agents head‐to‐head to clarify the drug‐specific heterogeneity noted throughout this review. Identifying which patients, most plausibly those with elevated baseline blood pressure and coexisting type 2 diabetes, stand to benefit most will be essential to positioning GLP‐1 receptor agonists within the broader strategy for cardiovascular risk reduction.

## Author Contributions

**Nora H. J. Alharbi:** writing – original draft, writing – review and editing, visualization, data curation, supervision, resources, project administration, software, methodology, conceptualization, investigation, formal analysis.

## Funding

The author has nothing to report.

## Consent

The author has nothing to report.

## Conflicts of Interest

The author declares no conflicts of interest.

## Data Availability

Data sharing is not applicable to this article, as no new data were generated or analyzed in this study. All information discussed in this review is drawn from previously published studies, which are cited in the text and listed in the reference list.
